# Supplementary material for: The interactome of intact mitochondria by cross-linking mass spectrometry provides evidence for coexisting respiratory supercomplexes
Source: Mol Cell Proteomics. 2017 Dec 8;17(2):216–32. doi: 10.1074/mcp.RA117.000470 (PMC5795388; doi:10.1074/mcp.RA117.000470)
Supplement: Supplemental Data [file supp_RA117.000470_133922_0_supp_23978_fzffwf.zip › spectra_annotation/mito_spectra_annotation/95-1-5-1-27-1.pdf]

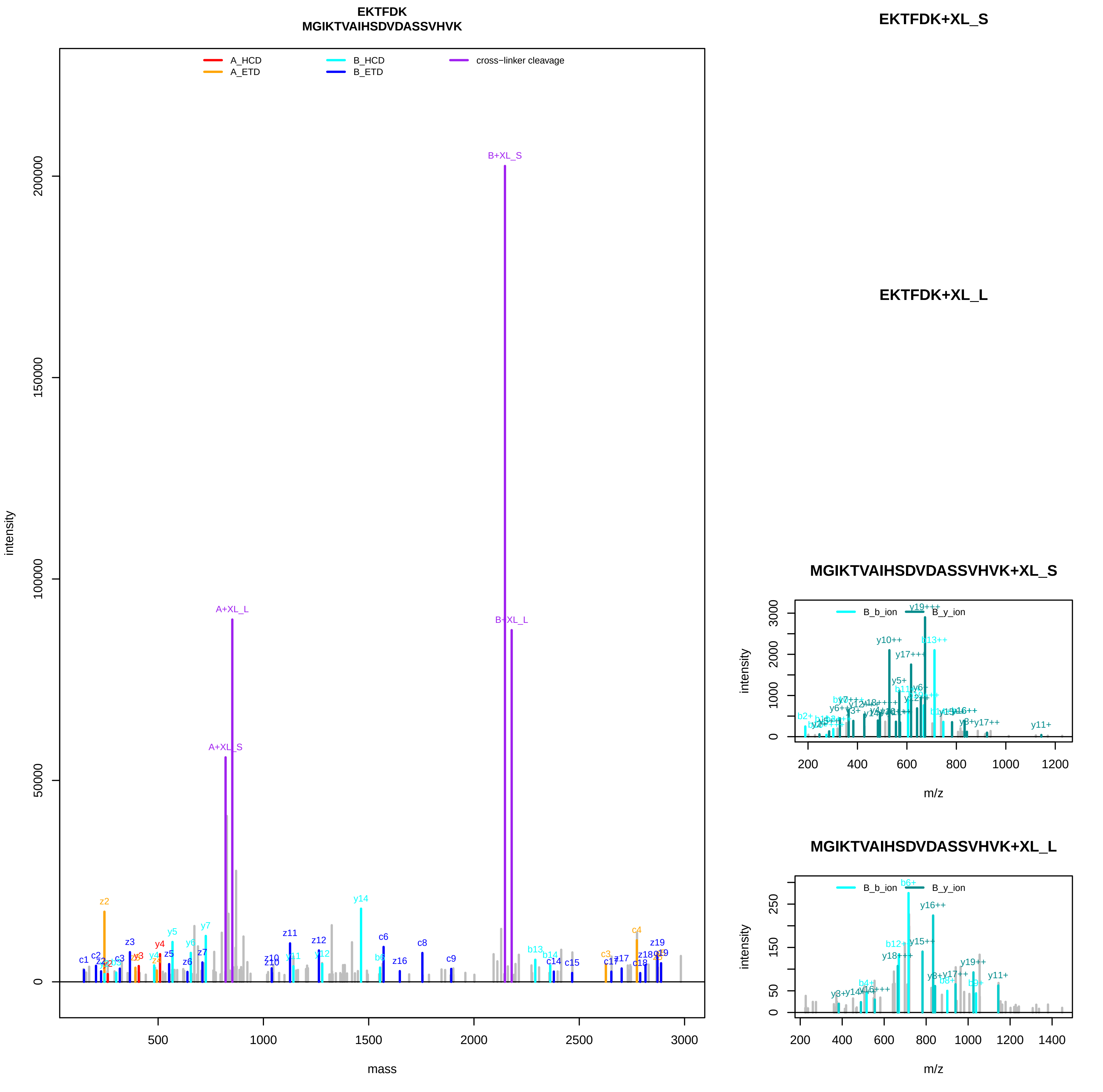

EKTFDK
MGIKTVAIHSDVDASSVHVK
A_HCD
A_ETD
B_HCD
B_ETD
cross−linker cleavage
B+XL_S
A+XL_L
B+XL_L
A+XL_S
intensity
mass
0
50000
100000
150000
200000
500
1000
1500
2000
2500
3000
y14
z11
z12
c6
c8
c9
z16
c4
z19
EKTFDK+XL_S
EKTFDK+XL_L
MGIKTVAIHSDVDASSVHVK+XL_S
B_b_ion
B_y_ion
y19+++
y10++
b13++
y17+++
y11+
intensity
m/z
MGIKTVAIHSDVDASSVHVK+XL_L
B_b_ion
B_y_ion
b6+
y16++
b12++
y15++
y18+++
y19++
y11+
intensity
m/z
